# PeSTK db a comprehensive data repository of Probiotic Serine Threonine kinases

**DOI:** 10.1038/s41597-022-01379-2

**Published:** 2022-06-08

**Authors:** Dhanashree Lokesh, Suresh PSN, Rajagopal Kammara

**Affiliations:** 1 Department of Protein Chemistry and Technology, CSIR-CFTRI, Mysore, 570020 India; 2 Computer Center Facility, Mysore, India; 3 Present Address: Center for Food Animal Health, The Ohio state University, Columbus, OH USA

**Keywords:** Bacteriology, Agriculture, DNA

## Abstract

The signal transduction pathway of prokaryotes involves a peptidoglycan synthesis cluster (PG) to sense external stimuli. One of the major components of the PG synthesis cluster is protein kinases (pknA - G). The sequence data of probiotic eSTKs (Eukaryotic like Serine, Threonine kinases) are obscure, scarce and essentially required to understand the role of probiotic microbes in combating infectious diseases. The most essential need to understand and develop certain therapeutic drugs against pathogens is the eSTK sequence data. Hence, we developed a comprehensive user-friendly data repository of probiotic eSTK’s (PeSTK), which holds 830 STK sequences. Therefore, the data resource of PeSTK developed is unique, an open-access very summative containing various probiotic eSTK’s in a single locality. The sequence datasets of the eSTK developed with easy-to-operate browsing as well as searching. Therefore, eSTK data resources should be useful for sequence-based studies and drug development. The sequence datasets are available at Figshare Digital Object Identifier/DOI of the sequences is 10.6084/m9.figshare.146606.

## Abstract (summary)

**Table:** 

Measurement(s)	Serine threonine protein sequences of probiotic microbes
Technology Type(s)	Softwares uploading and downloading of sequences from various public sources
Factor Type(s)	Serine threonine kinases of Probiotic microbes
Sample Characteristic - Organism	Bifidobacteria spp • Lactocobacillus spp • Enterococcus spp • Streptococcus spp • Leuconostoc spp • Pediococcus spp
Sample Characteristic - Location	India

## Background & Summary

Protein kinases, together with their associated phosphatases, play a significant role in signal transduction across both prokaryotes and eukaryotes, enabling them to quickly respond and adapt to constantly changing environments. To sense exterior inputs and control itself, the cell employs a well-established signal transduction pathway. To sense and regulate itself the cell follows a mechanism called signal transduction. Today, explicated bacterial genomic studies unveil the presence of serine/threonine kinases in bacteria is certain^1^ (Bakal and Davies, 2000). The term “eukaryote-like Ser/Thr kinases (eSTK)” refers to bacterial Ser/Thr kinases that have catalytic domains that are similar to those of eukaryotic Ser/Thr kinases. Irrespective of the host, eSTK’s are expanded in Gram-positive bacteria and proclaimed to play a significant role in signal transduction^1^. Besides the various functions of the eSTK (serine/threonine kinase) family plays a crucial role in contributing to bacterial pathogenesis partly in sensing the host environment and secondly by subverting the organism’s immune response^2^. As it intervenes in pathogenesis, eSTK’s are the second largest drug targets^3^. Studies on *Mycobacterial* kinases gave way for research in the area. The optimal growth of MTB depends only on three kinases namely pknA, pknB, and pknG out of 11 existing (Sassetti *et al*.)^4^ kinases. The constitutive expression of MTB pknA (protein kinase A) in *E. coli*^5^ and *in-vitro* inhibition studies of pknB^6^ in *Mycobacteria* revealed its contribution to the growth. Although pknA and pknB are present next to each other, they are independently essential for growth and survival of pathogen, if any loss of either of these individually or together alter the cell morphology^7^. Phosphorylation by kinases contributed to maintenance of morphology, and development of *Mycobacterium smegmatis*^8^ and *Myxococcus xanthus*^9^ respectively. A recent study on *Bifidobacteria* reported six kinases that were alike with all 12 Hank’s type conserved domains reported in *B. longum* and with varying degree of similarity in other *Bifidobacterial spp*^10^. Furthermore, evaluation of the *Bifidobacteria* kinases revealed genes pkb5 and pkb6 that are orthologous to pknB and pknA of MTB present in PG synthesis cluster^11^.

In recent years, around 150 kinase inhibitors have undergone clinical trials, and a few have been approved by FDA for human use^12^. The structural arrangement of pknA’s is conserved among prokaryotes, consisting of three domains beginning with the N-terminal domain, central kinase, and C-terminal domain. The pknA of *B. adolescentis* does not contain a transmembrane domain, a unique and major difference. Organisms like *Lactobacillus, Staphylococcus, Streptococcus*, and *Bacillus* are restricted to one or two serine/threonine kinases. Whereas other species such as *Mycobacteria, Nocardia*, and *Streptomyces* contain more than 10, 20, and 30 serine/threonine kinases, respectively^13^. A study on probiotic eSTK’s is essential to conserve the gut flora over inhibitors designed to target the pathogens. As mentioned earlier, the number of kinases varies among individual organisms, which might have a specific interacting partner for a distinct role^14^. To exploit it further, possessing the probiotic kinases on a single platform is necessary, and the data should be readily available at a single location and easily accessible. It was our concern to build the datasets containing only eSTK’s specific to probiotic organisms such as *Lactobacillus, Bifidobacteria, Enterococcus*, etc.

## Methods

**Fig. 1:**
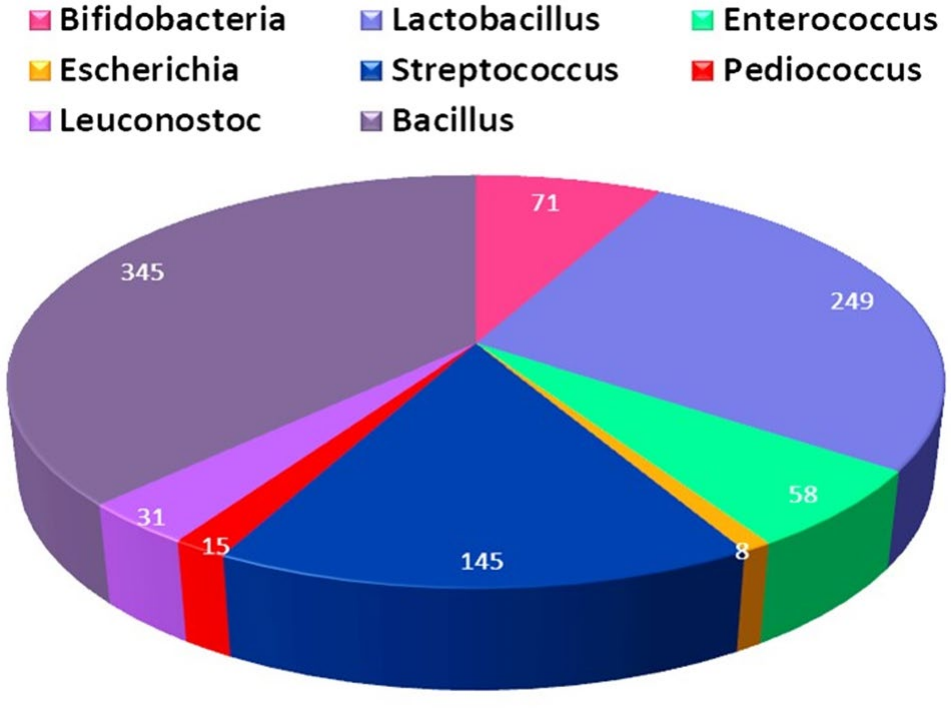
Pie chart showing the number of species present in the genera represented in a particular colour.

The overall population structure of eSTK’s of various probiotic microbes is shown in a pie chart in Fig. 1. The architecture and utility of eSTK datasets have been submitted with the following identification.

- The user will find the details of the eSTK protein like its Id, name, source, sequence characteristics.
- The eSTK sequences/datasets are made in an Excel sheet.

The available protein data and information/resources from SWISSPROT, Protein Information Resource (PIR)–Protein Sequence Database (PIR-PSD), NCBI, and UniProt were used. They were collected and further refined with data available in the public domain. All the sequences extracted were validated through their publications, references, and citations.

### PeSTKs domain architecture

The various online freely available software were followed to dissect, and understand the various domains of eSTK’s such as kinase domain, ATP, NP and Hank’s domain. We have identified domains and associated super families through analysis of PeSTKs employing the CD-Search tool^15^. In total, 299066 domains/super families were identified in 706 sequences out of 709. We have also catalogued different super families associated with individual sequences. Overall, 214 unique super families among 13135 redundant ones were recognized in PeSTK sequences. Further, we have curated 129 eukaryotic protein kinases using CDD string search^16^, which is used to find eukaryotic-like domains in PeSTK sequences. For each sequence, eukaryotic-like super families/domains were catalogued along with accession numbers and locations. We have identified 5285 eukaryotic-like domains/super families in 636 PeSTK sequences out of 706, which is mainly dominated by the PKc-like superfamily (cl21453).

### Kinase domain architecture and analysis

Overall, 709 protein sequences of probiotic eSTKs (Eukaryotic like Serine and Threonine Kinases) and prokaryotic kinases were first subjected to domain search employing the batch web CD-Search Tool (31777944, 25414356)^15^. In brief, an automatic search mode with an expected value threshold of 0.01 was implemented against the CDD-58235^16^ PSSMs database^17^, and composition-corrected scoring was enabled. To, further characterize the eukaryotic kinase domains in the sequences, the conserved domains database (CDD) (31777944, 25414356) was searched with the query “Eukaryotic protein kinases”. Further, using the in-house developed script, eukaryotic kinase domains were searched in the exhaustive domain list of proteins, and domain names, numbers, and locations (start-end) were reported explicit to PeSTKs.

### Hank’s domain architecture

The sequence alignment of the protein was performed using software MAFFT7^18,19^ with the default parameter adjusted. The catalytic domains of kinases from the aligned sequence was considered and identified Hank’s domain based on the previous publications^20–23^ and grouped using software Jalview^24^. The list of Hank’s domains sequences are available available at Figshare Digital Object Identifier/DOI of the sequences is 10.6084/m9.figshare.19930760.v1.

## Data Records

The sequence information about eSTKs was mined on various public databases like NCBI, Protein Information Resource (PIR) – Protein Sequence Database (PIR-PSD). UniProt, PDB, and Bio-Cyc databases were mined to extract eSTK information. The generated hits were registered into the dataset if i) they have conserved domains such as kinase domain, and ATP binding; ii) the amino acid sequences of eSTK have been elucidated; iii) they contain more than 100 amino acid residues. Searching the PubMed literature database yielded the literature reports.

Initially, relevant reports and sequences were collected using the keywords “Serine-Threonine Proteins”, “Probiotic Serine-Threonine Proteins/eSTKs of *Lactobacillus, Bifidobacteria”*, “Signaling molecules” and “Signaling Proteins”, which resulted in over 2500 kinases. The resultant sequences were further, screened for Hank’s-type domains. Subsequently, sequences were obtained scrutinized, and sorted out based on the validated reports, which narrowed down the entries to 800 sequences containing potential validated data. Review articles were preferred, but non-English articles such as Chinese, French, and Swiss were not. Precedence was given to articles with experimentally proven or validated sequences. The dataset contains 830 eSTK entries. The information in these datasets was classified as organism, species specificity, sequence ID, description, and reference. The datasets are available at Figshare Digital Object Identifier/DOI of the sequences is 10.6084/m9.figshare.19930760.v1^25^. The eSTK database is freely available for research and development at http://estkdb.cftri.res.in. The domain architecture and their eSTK components such as kinase domains, hanks domain sequence data for each probiotic microbe has been deposited in the Figshare as explained above. Hence, the datasets containing sequences are available at Figshare Digital Object Identifier/DOI of the sequences is 10.6084/m9.figshare.19930760.v1.

All the components of the dataset, such as the list of probiotic microbes, the number of eSTK’s present, their protein sequences, the name of the corresponding gene, source, and closely related spp are available and free for the public. The data resource begins with the organism, giving options such as *E. coli, Bacillus, Lactococcus, Leuconostoc, Pediococcus, Enterococcus*, and *Bifidobacteria*. Subsequently, after choosing the specific organism and selection of species specificity, gives the details such as ID, Entry, and whole details of the specific eSTK. The data is available in the Figshare as shared above. Data pertaining to *Bacillus* and *Streptococcus* does not exist in the database, and has yet to be uploaded.

The desired eSTK sequences were extracted from each peer-reviewed and printed/published article. Datasets have been arranged in the form of rows and columns using a Microsoft Excel sheet/file. Each column represents the sequence and characteristics of the eSTK’s. Data organization in brief; begins with ID, Entry, Entry name, Status, Protein, Gene name, organism, species, length, sequence, authors, journal, and society. Once the database/repository was made, subsequently all the sequences were made as separate files (organism based) they were put in Excel sheet.

The following fields describe the dataset:

1. UniProt ID: Unique identification number provided for each sequence.
2. Sequence: All amino acid sequences are alphabetical represented and displayed in fasta format.
3. All eSTK sequences are formatted in a standard/universal single-letter amino acid code.
4. Nomenclature: Protein designation of the respective literature cited.
5. Source: The pedigree of the protein, and bacteria name.
6. References: Given each sequence by showing the abstract of the article.

### Dataset construction

**Fig. 2:**
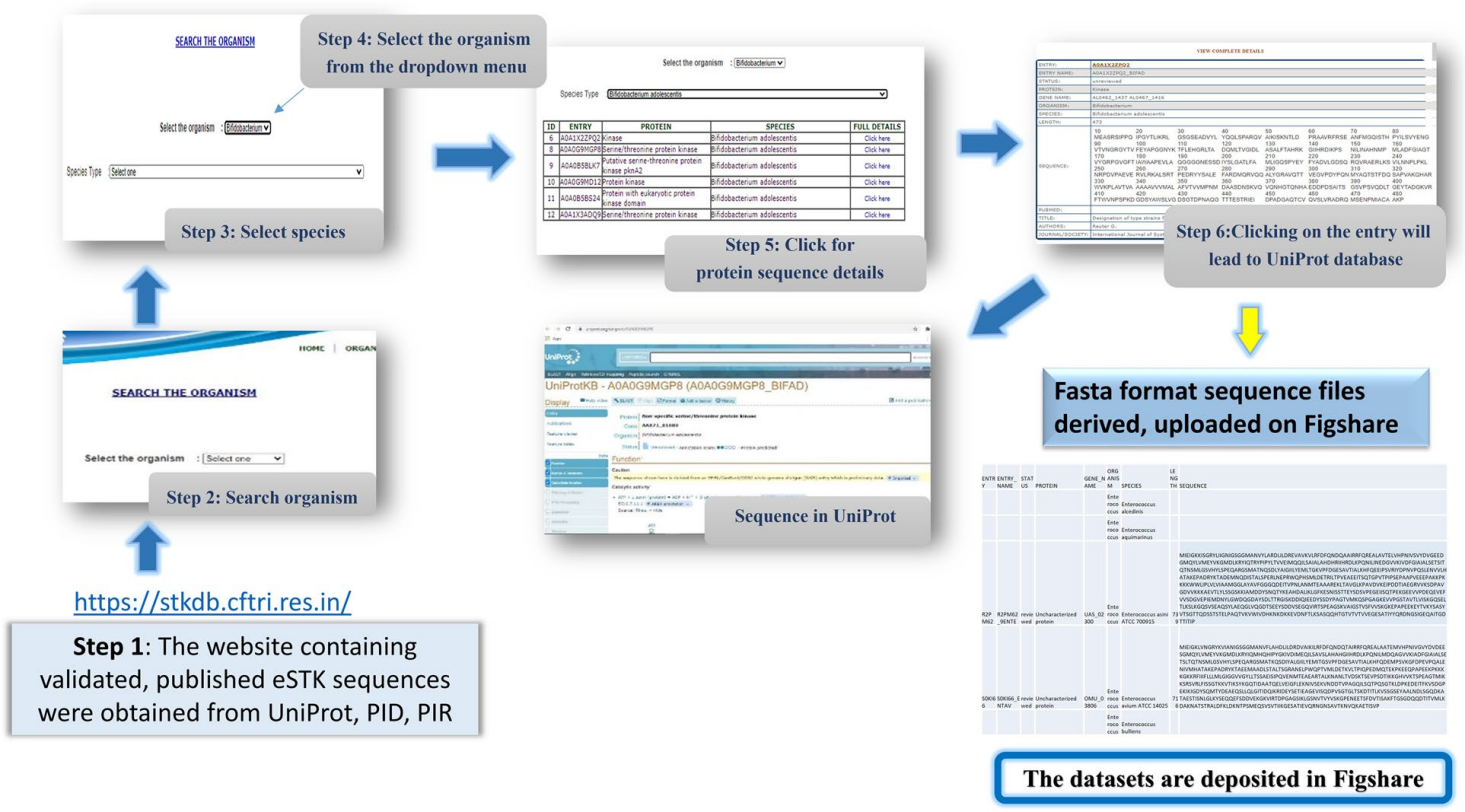
Architecture of datasets in Data descriptor.

In the data records section, the specific details of the eSTK sequence identification, isolation, and extraction are detailed. There are 830 eSTK entries in the dataset and information in this dataset is organized based on organism, species, sequence ID, description, reference, and UniProt link. The eSTK database was created using the obtained eSTK sequences, as shown in Fig. 2. Following that, sequences from the eSTK database were extracted converted into Fasta format and turned into bacteria-specific files. Finally, the sequences in Fasta format were submitted to the Figshare platform. Figure 2 depicts the data gathering, storage, and maintenance procedure in detail.

### Dataset details

The dataset currently contains the (eSTK) protein sequence information of eight probiotic spp, such as *Lactobacillus, Bifidobacterium, Enterococcus, Streptococcus, Pediococcus, Leuconostoc, Bacillus*, and *Escherichia coli*, which are approved, and extensively used as probiotic bacteria. Individual species genomes (figure 1) were obtained from publicly available database resources including the NCBI (http://ncbi.nlm.nih.gov/), BioCyc (https://biocyc.org/)^26^, UniProt (https://www.uniprot.org/). The DSMZ database eased our work on identifying several species reported to date, under the genus selected. Only the probiotic microbes, irrespective of their GRAS status, were taken into consideration for collecting the information on the kinase. Foremost, the kinases of particular species were dugout from the NCBI website and further confirmed by BLAST with UniProt. Irrespective of the status of the protein, the details from the page, including UniProt ID, entry name, entry ID, organism name, molecular weight, and UniProt link, were gathered in reference to the particular organism obtained by the DSMZ website and placed in the table containing the above details.

#### Data retrieval and utility

The PeSTK dataset is specific to probiotic organisms. Looking for an individual species will be easier, and the data will be recovered by researching, and selecting a specific species. Almost all probiotic eSTKs sequences are available in a single location, which increases their usefulness for structure-function studies and studies to understand develop investigated inhibitors. In addition, the data descriptor is also helpful for strategic cloning, expression in heterologous hosts, and subsequent protein purification, where protein sequence is a basic and essential requirement.

The PeSTK dataset provides a platform to investigate the kinases in detail to understand their sequence and their role in controlling various cellular functions. The kinases in human commensals are not as well-established as in pathogenic bacteria. Inhibitors targeting kinases have also been shown in studies to be promising antimicrobial agents in the future so learning about probiotics bacterial kinases would aid in the preservation of gut bacteria^27^. Since their discovery, plenty of eSTK’s have been reported, and data has been compiled based on the Hank’s and Hunter classification given in the kinG database^28^. Although almost all the eSTK sequences were published they are not readily available for research because of their dispersed nature. Therefore, building a PeSTK dataset to assess the diversity and distribution among probiotic strains (Fijan, 2014) helps future work gain an insightful understanding of the vital biological role of eSTK’s in an individual organism.

The dataset contains around 830 serine-threonine kinase sequences distributed among the various probiotic organisms. The details, including amino acid sequence, molecular weight, source, organism name, protein name, entry ID, and link to the UniProt, will give a detailed description of functional aspects. The probiotic kinase is the new and first approach to a better understanding of kinases and their role in regulating various cellular activities. The dataset represents all available and validated eSTK sequences, to some extent, even the PDB structures. We conclude that all the sequences and their information included in this dataset are referred to cited, and validated.

#### Data retrieval and export capability

The dataset is user-friendly, deposited in the public repository as directed by the scientific data journal editorial, and is mandatory for submission. The datasets are available at Figshare Digital Object Identifier/DOI of the sequences is 10.6084/m9.figshare.19930760.v1. Five different categories can be followed for browsing the dataset, viz. i) Protein name, or sequence ii) Organism, iii) Protein ID, iv) References, and v) STK specific information can all be retrieved by using the appropriate keywords. This facility serves to determine the appropriate information in a short period. The sequences were organized systematically so that they can be viewed, extracted, and copied for research purposes without any problem.

## Technical Validation

In summation of the above, the other fields such as structure and physicochemical properties are also included in the respective positions of the STK resource (http://stkdb.cftri.res.in) Charge, PI values. Amino acid compositions are presented automatically by linking the datasets to globally well-known protein sequence public databases such as UniProt and Pubmed (this can be accessed on our URL). No special efforts have been made to analyze the hydrophobic and hydrophilic nature of the proteins^29^. The stand-alone probiotic eSTK dataset presently contains 830 kinase sequences. Figure 1: Pie chart showing several species present in each genus represented by a particular color. The PDB structures are not available as many proteins are yet to be revised and insightful studies are needed. Datasets are deposited in the Figshare portal in accordance with the scientific data editorial. They are placed in an organized, systematic manner where there is a single file containing all the sequences as stated. The file contains the data sequence, their ID, entry, and name as eSTK protein, followed by the species they belong to, and enclosing full details. The full details section shows the gene name, species, length of the sequence, and finally, alphabetically, the amino acid sequence. They also give PubMed details, the title of the manuscript, the author, and the journal name. This information helps in validating the sequences and their authenticity.

The various domains of serine-threonine kinases, such as Hank’s and kinase domains were arranged in an excel sheet. The resulting files are available at Figshare Digital Object Identifier/DOI of the sequences is 10.6084/m9.figshare.19930760.v1 representing domains and associated super families of PeSTKs, different super families associated with individual sequences. Followed by unique super families of PeSTKs, eukaryotic-like domains/super families of PeSTKs. Finally, Hank’s domains found in PeSTKs of *Bifidobacteria, Lactococcus, Leuconostoc, E. coli*, and *Enterococcus spp*.

## Usage Notes

The eSTK dataset contains interfaces such as protein sequences and searches, organisms. This implies that any probiotic organism can be easily found by getting in. This further takes us to selecting the specific spp, finally taking us to the sequences and linking to UniProt. A large number of eSTK sequences, their source and the data validation are available in one single dataset at a single location that makes it easily accessible and convenient for usage. This makes the dataset unique for eSTK research to develop drugs or inhibitors or even to understand the action and mechanism of these proteins to sense, the external environment. Hence, there will be a lot of usage of this dataset/data descriptor for research. In future, we intend to update the Figshare records as well, and when updating the data in the PeSTKdb website.

All of the kinases possess the protein kinase “signature” motifs, including 11 conserved subdomains as per Hank’s criteria. Amino acid sequence alignment of these STPK family members revealed that 15 catalytically important residues were conserved across all of them. We used two databases and software to figure out how many eukaryotic like domains exist in kinase. In summary, we conclude that almost all probiotics considered in the study possess maximum 12 Hank’s domains, and 17 kinase domains. There are a few *Bifidobacterial, Lactobacillus*, and *Leuconostoc* do not contain them. *E. coli* is the only organism that does not have Hank’s domains.

## Data Availability

No custom code was used in the data acquisition of these datasets.
